# Rapid activation of ARF6 after RAF inhibition augments BRAF^V600E^ and promotes therapy resistance

**DOI:** 10.1038/s41388-026-03805-w

**Published:** 2026-04-28

**Authors:** Junhua Wang, Yinshen Wee, Thomas Jacob, Aaron Rogers, Lise. K. Sorensen, Deja M. Brooks, Prachi Gupta, Joshua K. H. Tay, Emily C. Wilson, Tong Liu, Eric A. Smith, YN Vashisht Gopal, Michael A. Davies, Martin McMahon, Sheri L. Holmen, Robert L. Judson-Torres, Roger K. Wolff, Allie H. Grossmann

**Affiliations:** 1 Department of Oncologic Sciences, University of Utah, Salt Lake City, UT USA; 2 Department of Pathology, University of Utah, Salt Lake City, UT USA; 3 School of Dentistry, College of Oral Medicine, Taipei Medical University, Taipei, 110 Taiwan; 4 Providence Cancer Institute, Earle A. Chiles Research Institute, Portland, OR USA; 5 Department of Dermatology, University of Utah, Salt Lake City, UT USA; 6 Department of Melanoma Medical Oncology, The University of Texas MD Anderson Cancer Center, Houston, TX USA; 7 Huntsman Cancer Institute, University of Utah, Salt Lake City, UT USA; 8 Department of Surgery, University of Utah, Salt Lake City, UT USA

**Keywords:** Targeted therapies, Melanoma

## Abstract

The intrinsic ability of cancer cells to evade death underpins tumorigenesis, progression, metastasis, and the survival of drug-tolerant persister (DTP) cells. Herein, we discovered that the small GTPase ARF6 plays a central role in tumor survival by fortifying RAF oncoprotein levels. ARF6 activation was sufficient to increase BRAF^V600E^, ARAF, and CRAF proteins through a post-transcriptional mechanism, while sustained inhibition of ARF6 eventually led to decay. In a genetically engineered model of aggressive melanoma, tumor-specific *Arf6* deletion attenuated BRAF^V600E^ protein expression and MAPK signaling and prevented rapid tumor progression. In human melanoma cells, pharmacologic inhibitors of BRAF^V600E^ uniformly induced swift activation of ARF6, driving a positive feedback loop that restored MAPK-driven anti-apoptotic signaling and supported drug-tolerant survival and growth. Furthermore, in patient-derived melanoma xenografts with innate or clinically acquired resistance to MAPK inhibitors, ARF6 silencing alone significantly suppressed tumor growth in vivo. When combined with BRAF and MEK targeted therapy in vitro, inhibition of ARF6 markedly reduced survival and drug-tolerant growth. Collectively, these findings reveal a previously unknown mechanism of maintaining BRAF^V600E^ protein expression that preserves the MAPK pathway during targeted therapy. This ARF6-dependent mechanism may be exploited in BRAF^V600E^ driven cancers as a therapeutic vulnerability.

## Introduction

Avoiding cell death is fundamental to the progressive acquisition of hallmark cancer behaviors [1], and to the survival of drug tolerant persister (DTP) cells that give rise to therapy resistance [2]. Proposed origins of DTP cells include clonal selection of pre-existing drug-resistant cells, and drug-induction of a reversible DTP state that enables the outgrowth of fixed resistance [3, 4]. The molecular mechanisms of DTP cell emergence are complex and incompletely understood.

Mitogen activated protein kinase (MAPK) signaling is hyperactivated in many cancer types and directly opposes cell death by inactivating pro-apoptotic proteins of the intrinsic pathway [5]. In cutaneous melanoma, where most cases harbor somatic mutations in *BRAF, NRAS*, or *NF1* that cause aberrant MAPK signaling [6], resistance to MAPK targeted therapy remains a significant clinical challenge [7].

We detected abnormally elevated levels of ARF6-GTP, the active form of the small GTPase ARF6, in melanoma [8]. ARF6 activation in melanoma can occur through extracellular signals such as HGF [9, 10], WNT5a [11], and Interferon-γ [12]; or by altered expression of guanine exchange factors (GEFs) or GTPase activating proteins (GAPs) [8, 12, 13]. HGF and WNT5a signaling also mediate resistance to MAPK targeted therapy in melanoma [14–17]. We showed that ARF6-GTP promotes tumor development, progression, and acceleration of metastasis in murine models of BRAF^V600E^ melanoma [8, 11, 12]. Functionally, ARF6 activation enhances tumor cell invasion and adaptive immune suppression [8, 9, 11, 12]. Hence, ARF6 mediates the acquisition of at least two hallmark malignant behaviors [1]. Whether ARF6 has a role in DTP cell survival is unknown.

ARF6 is a ubiquitously expressed protein critical for endomembrane trafficking and actin cytoskeleton remodeling [18] and has diverse physiologic roles across multiple organ systems [19–30]. To expand our understanding of ARF6 function in cancer, we interrogated proteomic alterations induced by ARF6 activation and discovered that ARF6 dynamically regulates expression of the BRAF oncoprotein in melanoma and other cancer types, impacting tumor cell survival, including during MAPK targeted therapy.

## Results

### ARF6 augments a dynamic pool of oncogenic BRAF protein

**Fig. 1:**
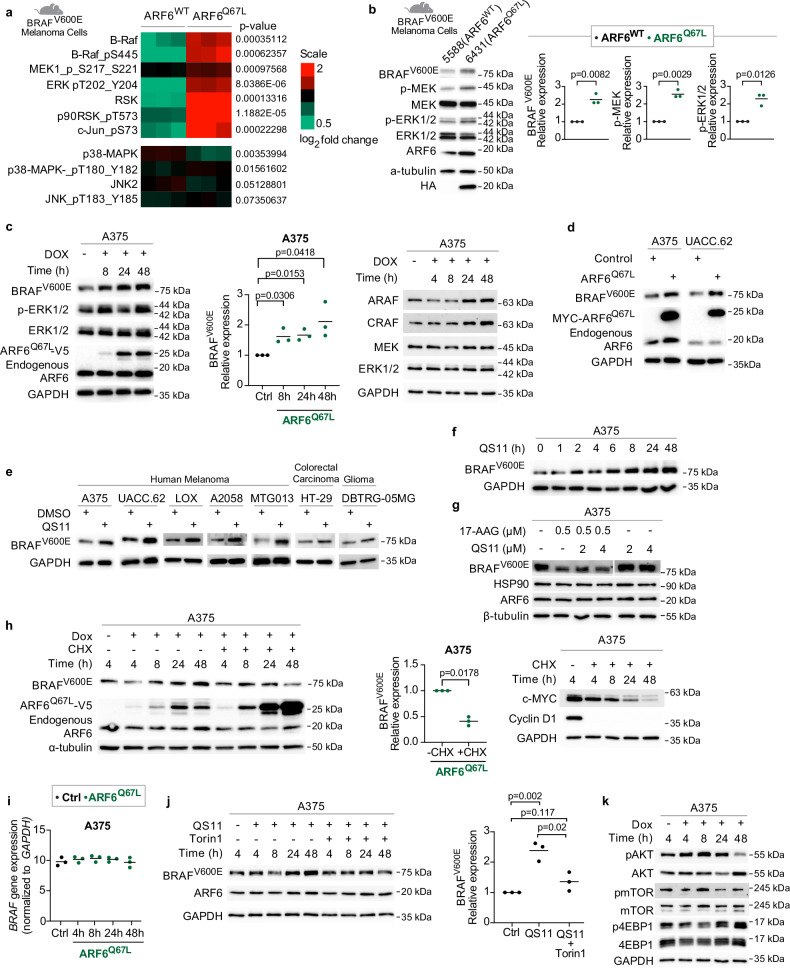
ARF6 is sufficient to control oncogenic BRAF protein levels through protein translation. **a** Relative amount of MAPK signaling proteins in tumor cells derived from *Braf*^*V600E*^*; Cdkn2a*^*f/f*^*; Arf6*^*WT*^ or *Braf*^*V600E*^*; Cdkn2a*^*f/f*^*; Arf6*^*Q67L*^ mice, detected by Reverse Phase Protein Array, two-tailed t-test. n = 3 replicates per cell line. **b**–**h**, **j**, **k** Western Blot for indicated proteins**. b** Murine melanoma cells derived from *Braf*^*V600E*^*; Cdkn2a*^*f/f*^*; Arf6*^*WT*^, or *Braf*^*V600E*^*; Cdkn2a*^*f/f*^*; Arf6*^*Q67L*^ mice., n = 3 biological independent experiments. **c** Human A375 melanoma with doxycycline (DOX)-inducible ectopic expression of ARF6^Q67L^, BRAF^V600E^ Western blot n = 3 biological independent experiments, h = hours. **d** Human UACC.62 and A375 cells with or without adenoviral-mediated ectopic expression of ARF6^Q67L^, control= empty vector. **e** 4 μM QS11 for 48 h in human A2058 melanoma, HT-29 colorectal carcinoma, and DBTRG-05MG glioma. 2 μM QS11 for 24 h in other cell lines. **f** 2 μM QS11, h = hours. **g** 17-AAG and QS11 for 24 h in A375 cells. **h** 20 μg/ml cycloheximide (CHX) in A375 cells with doxycycline (Dox)-inducible ectopic expressed ARF6^Q67L^. BRAF^V600E^ protein quantification at 48 h., n = 3 biological independent experiments. **i** Quantitative RT-PCR for *BRAF* mRNA in A375 cells with doxycycline (Dox)-inducible ectopic expressed ARF6^Q67L^, n = 3 biological independent experiments, h=hours. **j** 4 μM QS11 and 250 nM Torin 1 in A375 cells. BRAF^V600E^ protein quantification at 48 h, n = 3 biological independent experiments. **k** A375 cells with doxycycline (Dox)-inducible ectopic expressed ARF6^Q67L^. h = hours. **b**, **c**, **h**, **j** Two-tailed ratio paired t-test.

In early passage murine melanoma cell lines with homozygous *BRAF*^V600E^ mutation, derived from our genetically engineered murine melanoma models [8, 12], proteomic analysis showed higher levels of BRAF^V600E^ protein, and increased phosphorylated MEK1, ERK, RSK and Jun, in cells expressing constitutively active ARF6-GTP (ARF6^Q67L^) compared to ARF6^WT^ (Fig. 1a). In contrast, p38 MAPK-JNK signaling was unaltered (Fig. 1a). ARF6-GTP-induced BRAF^V600E^ expression was confirmed by Western blot (Fig. 1b). These findings align with our previously published genomic data from this tumor model showing upregulation of genes in the MAPK cascade in bulk tumor transcriptomes [8]. Based on these findings, we hypothesized that ARF6 controls MAPK signaling by regulating oncogenic BRAF expression. In pursuit of this, we interrogated human melanoma cells and found that doxycycline-induced, ectopically expressed ARF6-GTP, in the form of ARF6^Q67L^ (Fig. S1a), or adenoviral delivered ARF6^Q67L^, augmented endogenous BRAF^V600E^ expression in human melanoma cells (Fig. 1c–d). Endogenous levels of ARAF, CRAF and phosphorylation of ERK rose in parallel with BRAF^V600E^ (Fig. 1c). Consistent with genetic activation of ARF6, pharmacological activation of ARF6 with QS11 (Fig. S1b), an inhibitor of ARF GTPase Activating Protein 1 [31], increased BRAF^V600E^ protein expression in human melanoma, colorectal carcinoma, and glioma cell lines (Fig.1e, S1c). BRAF^V600E^ protein levels rose quickly after treatment with the ARF6 agonist QS11, as early as two hours, and continued to accumulate over 48 h (Fig. 1f). Wild-type, endogenous BRAF protein increased in a similar manner in SKMel2 human melanoma cells (Fig. S1d). These data demonstrate that sustained ARF6 activation is sufficient to acutely increase endogenous RAF proteins.

The BRAF^V600E^ oncoprotein is stabilized by the chaperone protein HSP90 [32, 33], limiting proteasome-mediated degradation. Consistent with this, BRAF^V600E^ was stable in the presence of cycloheximide for at least 24 h (Fig. S1e), whereas decay was readily observed within this time frame when cells were treated with the HSP90 inhibitor 17-AAG (Fig. S1f). These data confirm that HSP90 prolongs the half-life of the BRAF oncoprotein. Unlike HSP90, ARF6 prevents lysosome mediated degradation of proteins through endosomal recycling [12, 34, 35]. Thus, we asked if BRAF^V600E^ might be degraded by the lysosome. Blocking lysosomal degradation by Bafilomycin A1 or DC661 failed to increase BRAF^V600E^ protein (Fig. S1g). Thus, it is unlikely that ARF6 regulates oncogenic BRAF expression through endolysosomal trafficking. Activation of ARF6 did not alter HSP90 protein expression (Fig. S1h), nor did it rescue BRAF^V600E^ from degradation during 17-AAG treatment (Fig. 1g), when the oncoprotein is vulnerable to proteasome degradation. Taken together, these data suggest that ARF6 does not regulate machinery controlling RAF degradation. Interestingly, inhibition of protein translation with cycloheximide prevented the accumulation of BRAF^V600E^, ARAF, and CRAF proteins upon ARF6 activation (Fig. 1h and S1i). Activation of ARF6 failed to alter *BRAF* mRNA levels in A375 melanoma cells, which harbor a homozygous *BRAF*^V600E^ mutation (Fig. 1i), demonstrating that ARF6-mediated upregulation of BRAF^V600E^ occurred without altering *BRAF* oncogene expression.

Because ARF6 has been reported to potentiate mTOR signaling [8, 36, 37], we hypothesized that ARF6-GTP mediated BRAF^V600E^ expression could be mTOR dependent. Inhibition of mTOR activity with Torin 1 prevented the boost in BRAF^V600E^ protein induced by QS11 (Fig. 1j), supporting that ARF6 activation might augment mTOR-mediated protein translation. Consistent with this, ARF6-GTP increased phosphorylation of 4EBP1 in human melanoma cells (Fig. 1k). Overall, these data confirm that ARF6 activation is sufficient to increase BRAF^V600E^ protein levels and raise the possibility that ARF6 regulates mTOR-dependent protein translation.

### ARF6 is necessary for maintenance of the BRAF^V600E^ protein

**Fig. 2:**
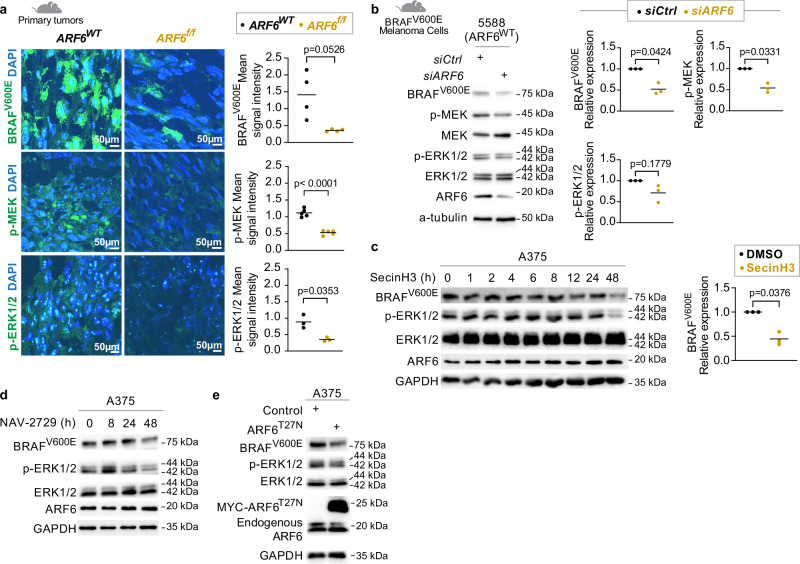
ARF6 is necessary to maintain oncogenic BRAF protein levels. **a** Representative immunofluorescence images of cryo-embedded frozen tumor tissues from *Braf*^*V600E*^*; Cdkn2a*^*f/f*^*; Arf6*^*WT*^ or *Braf*^*V600E*^*; Cdkn2a*^*f/f*^*; Arf6*^*f/f*^ mice, ×1200 magnification. Two-tailed unpaired *t*-test. **b**–**e** Western blot for indicated proteins. **b** Murine melanoma cells derived from *Braf*^*V600E*^*; Cdkn2a*^*f/f*^*; Arf6*^*WT*^ mice, transiently transfected with siRNAs, *n* = 3 biological independent experiments. **c** Human A375 melanoma cells treated with 10 μM SecinH3, BRAF^V600E^ protein quantification at 48 h, n = 3 biological independent experiments. **d** Human A375 melanoma cells treated with 5 μM NAV-2729. **e** Human A375 cells with or without adenoviral-mediated ectopic expression of ARF6^T27N^, control= empty vector. **b**, **c** Two-tailed ratio paired *t*-test.

In contrast to ARF6 activation, deletion of *Arf6* in BRAF^V600E^ murine melanoma tumors [12] reduced total BRAF^V600E^ levels and downstream phosphorylated MEK (p-MEK) and ERK (p-ERK), detected by immunofluorescence in situ (Fig. 2a and S1j). Consistently, silencing of *Arf6* downregulated BRAF^V600E^ and p-MEK detection in murine melanoma cells (Fig. 2b). To test whether inactivation of ARF6 (ARF6-GDP) could produce the same effect in human melanoma, we treated A375 cells with SecinH3, an ARF6 guanine exchange factor inhibitor that reduces ARF6-GTP levels [11, 38] (Fig. S1k) and reduces spontaneous metastasis of human BRAF^V600E^ melanoma xenograft tumors [11]. In human melanoma cells, SecinH3 significantly reduced BRAF^V600E^ protein within 48 h of treatment (Fig. 2c). NAV-2729, a direct inhibitor of ARF6 GTPase function [13] and ARF GEFs and GAPs [39] (Fig. S1l), also reduced BRAF^V600E^ protein after 48 h (Fig. 2d). Because SecinH3 and NAV-2729 are not highly specific inhibitors of ARF6, we tested ectopic expression of a dominant-mutant interfering form of ARF6, ARF6^T27N^, which reduced ARF6-GTP (Fig. S1m) and BRAF^V600E^ protein (Fig. 2e), suggesting that ARF6 activation may be necessary to maintain expression of endogenous BRAF^V600E^. Importantly, the kinetics of endogenous BRAF^V600E^ decay, between 24 and 48 h after SecinH3 or NAV-2729 treatment, was identical to cycloheximide (Fig. S1e). Together these data demonstrate that ARF6 may be necessary to maintain steady state levels of the BRAF^V600E^ oncoprotein and suggest that targeted inhibition of ARF6 might be an alternative approach to reducing BRAF^V600E^ oncoprotein expression.

### ARF6-GTP promotes tumor survival by protecting against apoptosis

**Fig. 3:**
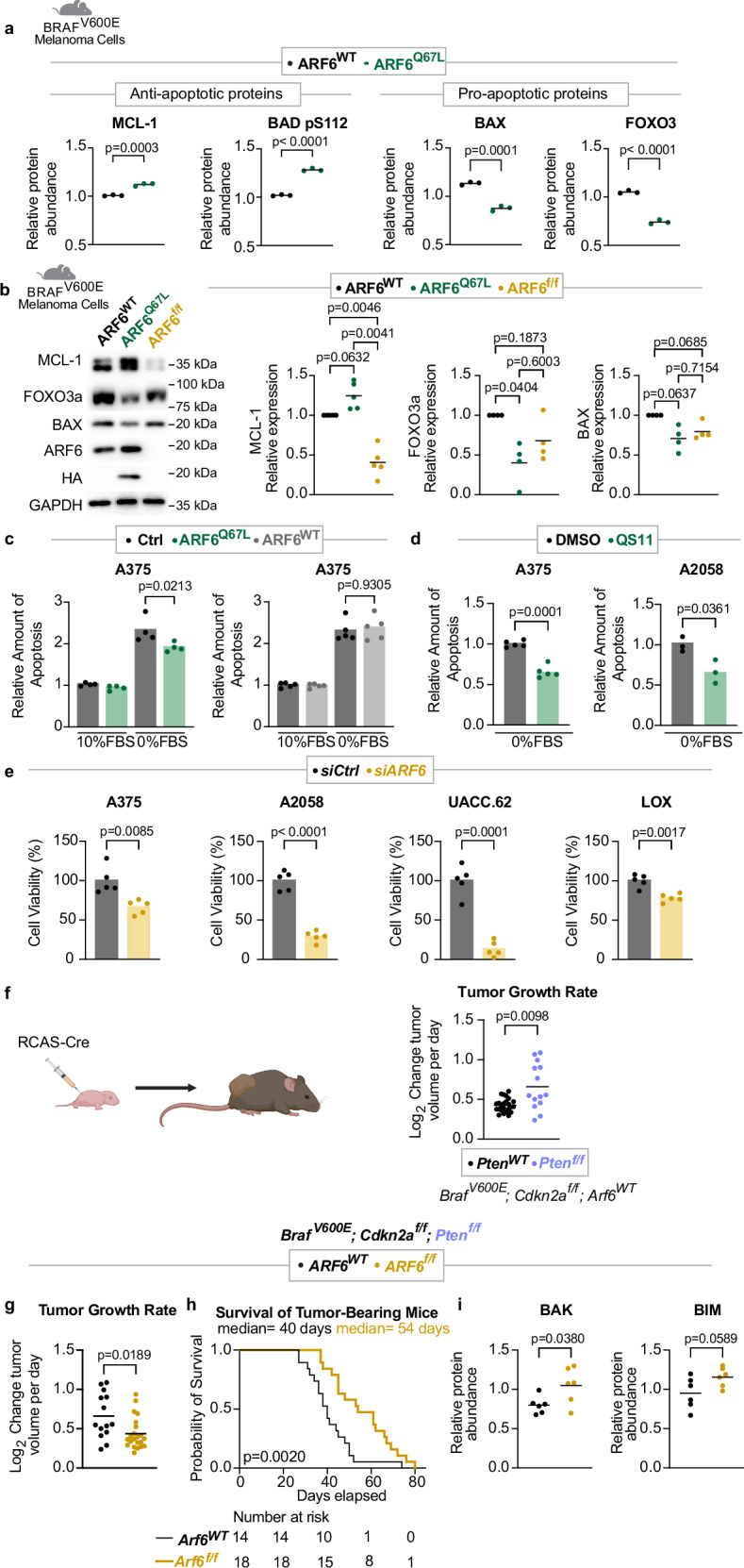
ARF6 promotes tumor survival and accelerated disease progression. **a** Apoptotic protein profile of tumor cells derived from *Braf*^*V600E*^*; Cdkn2a*^*f/f*^ mice detected by Reverse Phase Protein Array. Two-tailed t-test, n = 3 replicates per cell line. **b** Western Blot for indicated proteins in murine melanoma cells derived from *Braf*^*V600E*^*; Cdkn2a*^*f/f*^ mice. Two-tailed ratio paired t-test, n = 3 biological independent experiments. **c** Apoptosis detection, measured at 48 h, dox-inducible ectopic expressed ARF6^WT^ and ARF6^Q67L^ in A375 cells. One-way ANOVA with multiple comparisons, n = 4 replicates per condition. **d** Apoptosis detection, 4 μM QS11, measured at 48 h. Two-tailed unpaired t-test, n = 5 for A375 and n = 3 for A2058 replicates per condition. **e** Cell viability detection, measured at 72 h. Two-tailed unpaired t-test, n = 5 replicates per condition. **f** Rate of tumor growth measured from the time of initial detection in *Braf*^*V600E*^; *Cdkn2a*^*f/f*^; *Arf6*^*WT*^ mice. Two-tailed t-test with Welch’s correction, n = 24 *Pten*^*WT*^, n = 14 *Pten*^*f/f*^ mice. **g** Rate of tumor growth measured from the time of initial detection in *Braf*^*V600E*^; *Cdkn2a*^*f/f*^; *Pten*^*f/f*^ mice. Two-tailed t-test with Welch’s correction, *n* = 14 *Arf6*^*WT*^, *n* = 22 *Arf6*^*f/f*^ mice. **h** Survival of mice (before primary tumor reached 2 cm) after Cre injection (day 0) within 130 days, *n* = 14 *Arf6*^*WT*^, *n* = 18 *Arf6*^*f/f*^ mice, Log-rank (Mantle-Cox) test. Solid line within data points = mean. **i**, Apoptotic protein profile of whole tumors from *Braf*^*V600E*^; *Cdkn2a*^*f/f*^; *Pten*^*f/f*^ mice (n = 6 mice per group) detected by Reverse Phase Protein Array, two-tailed t-test.

Because MAPK signaling opposes the intrinsic apoptotic signaling pathway [5], we reasoned that ARF6-mediated fluctuations in BRAF^V600E^protein might be linked to survival. Proteomic clues to ARF6-mediated survival were evident in murine melanoma cell lines cultured in full serum (Fig. 3a, b). Compared to ARF6^WT^, cells expressing ARF6^Q67L^ showed significantly increased levels of the anti-apoptotic protein MCL-1 and phosphorylation (inactivation) of BAD at residue S112 (pS112) [5], as well as decreased levels of pro-apoptotic proteins BAX and FOXO3 (Fig. 3a). ARF6 dependent expression of MCL-1 and FOXO3 were confirmed by Western blot (Fig. 3b). ERK signaling has been reported to increase MCL-1 [40] and decrease FOXO3 [41] protein levels [5]. Thus, our data suggest that ARF6 activation might promote tumor cell survival through ERK-mediated anti-apoptotic signaling.

To test whether ARF6 activation could protect against apoptosis, we deployed a doxycycline-inducible system to express either ectopic ARF6^Q67L^ or ARF6^WT^ in human melanoma cells (Fig. S1a and S2a). Doxycycline alone did not alter viability of A375 parental cells, (Fig. S2b), while doxycycline-induced ARF6^Q67L^ significantly reduced apoptosis caused by serum withdrawal (Fig. 3c). In contrast, doxycycline-induced ectopic expression of ARF6^WT^ did not alter apoptosis caused by serum withdrawal (Fig. 3c), suggesting that the active form of ARF6 is required for the survival benefit. Consistent with ARF6^Q67L^, pharmacological activation of ARF6 with QS11 protected against apoptosis caused by serum starvation (Fig. 3d). QS11 alone failed to alter cell viability during steady-state conditions, when cells were cultured in full serum (Fig. S2c), indicating that the compound does not stimulate proliferation. Overall, these data demonstrate that ARF6 activation can protect against apoptosis during growth signal deprivation.

Given that ARF6 can regulate both PI3K-AKT [8] and BRAF^V600E^ -MAPK signaling (Fig. 1) and apoptosis upon serum withdrawal (Fig. 3c, d), we asked if ARF6 supports the viability of BRAF-mutant human cancer cells grown in full serum. Consistent with this, *ARF6* silencing led to significantly reduced viability in multiple human melanoma cell lines (Fig. 3e). Similarly, treatment with NAV-2729, a direct inhibitor of ARF6 GTPase function [13], reduced ARF6-GTP levels (Fig. S1l) and decreased cell viability in most of the human melanoma cells tested (Fig. S2d), although not as effectively as *ARF6* silencing (Fig. 3e). These data demonstrate that ARF6 can optimize survival during normal growth conditions.

### ARF6 is required for accelerated tumor progression caused by PTEN loss

In parallel with the MAPK pathway, survival signaling can also originate from the PI3K-AKT pathway [42] and we previously reported that activation of ARF6 enhanced PI3K expression and PI3K-AKT signaling [8]. *PTEN* loss of function mutations activate the PI3K-AKT pathway, are frequently detected in cutaneous melanoma [6], cooperate with mutant BRAF or NRAS to drive melanomagenesis [43, 44], and accelerate primary tumor growth in genetically engineered *Dct::TVA, Braf*^*V600E*^*; Cdkn2a*^*flox/flox*^ murine melanoma models induced in epidermal melanocytes of the ear pinnae [45]. Like pinnae tumors, deletion of *Pten* dramatically accelerated the growth of BRAF^V600E^ melanoma induced in the flank (Fig. 3f). To test the necessity of ARF6 in this highly aggressive model, we crossed *Arf6*^*flox/flox*^ (*Arf6*^*f/f*^) mice with the *Dct::TVA, Braf*^*V600E*^*; Cdkn2a*^*f/f*^*; Pten*^*f/f*^ mice. In this model, tumor-specific loss of *Arf6* (Fig. S2e) significantly reduced tumor growth to a level equivalent to *Pten*^*WT*^ tumors (measured from the time of tumor formation, Fig. 3g), and prolonged overall survival despite the absence of PTEN (Fig. 3h). Unlike *Pten*^*WT*^ mice [12], loss of ARF6 did not reduce overall tumor incidence in *Pten*^f/f^ mice (Fig. S2f), demonstrating that loss of PTEN is sufficient to overcome the weakened tumor initiation phenotype we previously observed with *Arf6* knockout. Nevertheless, loss of ARF6 significantly delayed tumor onset in *Pten*^f/f^ mice (Fig. S2f). Consistent with the *Pten*^*WT*^ tumor cell lines (Fig. 3b), tumors from *Pten*^f/f^; *Arf6*^f/f^ mice showed increased levels of pro-apoptotic proteins BAK and BIM (Fig. 3i), suggesting enhanced apoptosis signaling in the absence of ARF6. Given that *Arf6* deletion prevented primary tumor acceleration caused by PTEN loss (Fig. 3f, g), there is a component of ARF6-dependent survival that is necessary for, and/or functions independently of the PI3K pathway. Indeed, ARF6-dependent survival may also originate from rheostatic control of RAF expression (Figs. 1–2) and downstream, MAPK-mediated anti-apoptotic signaling.

### ARF6 is activated by RAF inhibition, protects against MAPK inhibitor-induced apoptosis, and potentiates resistance to MAPK inhibition

**Fig. 4:**
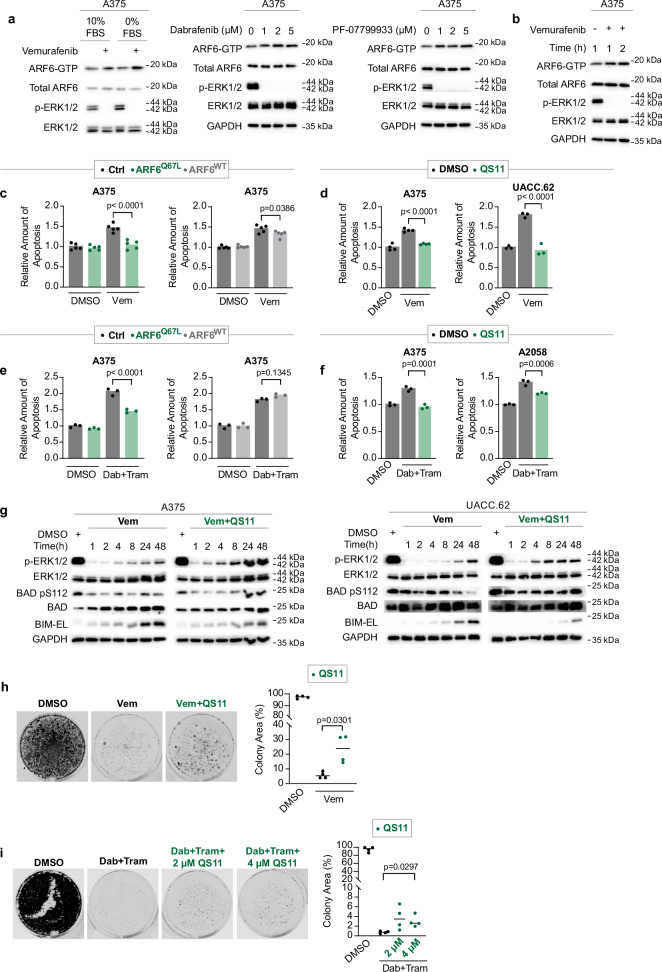
ARF6 activation protects against MAPKi-induced apoptosis and promotes the development of MAPKi-resistance cells. **a**, **b** Total ARF6 and ARF6-GTP pulldown in A375, 5 μM vemurafenib for 4 h or as indicated, Dabrafenib treatment for 4 h, PF-07799933 treatment for 2 h in 0% FBS media. **c**–**f** Apoptosis detection. One-way ANOVA with multiple comparisons. **c** 1 μM Vemurafenib, dox-inducible ectopic expressed ARF6^WT^ and ARF6^Q67L^ in A375, apoptosis measured at 48 h, Ctrl= no doxycycline, n = 5 replicates per condition. **d** 1 μM Vemurafenib, 4 μM QS11 for A375, n = 4 replicates per condition, apoptosis measured at 48 h, 2 μM Vemurafenib, 4 μM QS11 for UACC.62, n = 3 replicates per condition, apoptosis measured at 24 h. **e** 1.25 μM Dabrafenib, 0.0625 μM Trametinib, dox-inducible ectopic expressed ARF6^WT^ and ARF6^Q67L^ in A375, apoptosis measured at 48 h, Ctrl= no doxycycline, n = 3 replicates per condition. **f** 1.25 μM Dabrafenib, 0.0625 μM Trametinib, 4 μM QS11, apoptosis measured at 48 h, n = 3 replicates per condition. **g** Western Blot for indicated proteins. 1 μM Vemurafenib, 4 μM QS11 in A375. 2 μM Vemurafenib, 4 μM QS11 in UACC.62. **h**, **i** Colony outgrowth assay in A375. Two-tailed unpaired t-test. n = 4 biological independent experiments. **h** 1 μM Vemurafenib, 4 μM QS11, for 30 days. **i** 250 nM Dabrafenib, 12.5 nM Trametinib, 2 μM QS11, 4 μM QS11, for 30 days.

Because ARF6 can regulate RAF protein expression (Figs. 1–2), we asked if BRAF inhibition alters ARF6 activation. Remarkably, class I BRAF inhibitors, vemurafenib or dabrafenib, increased ARF6-GTP levels (Fig. 4a). This occurred both in the presence and absence of serum and is reproducible in independent BRAF^V600E^ cell lines (Fig. 4a and S3a). In contrast to ARF6, ARF1-GTP remained constant with dabrafenib treatment (Fig. 4a and S3b). Notably, the pan-mutant BRAF inhibitor PF-07799933, which inhibits BRAF mutant monomers and dimers and has antitumor activity in treatment refractory patients [46], also increased ARF6-GTP levels in human melanoma (Fig. 4a). Importantly, ARF6 activation occurred rapidly after BRAF inhibition, as early as one hour (Fig. 4a, b), suggesting that ARF6 activation functions in an acute adaptive response pathway to BRAF-targeted therapy.

Because ARF6 was rapidly activated upon RAF inhibition and ARF6-GTP promoted survival upon serum withdrawal (Figs. 4a, b, 3c, d), we asked whether ARF6 activation can facilitate survival during MAPK inhibitor (MAPKi) treatment. Indeed, genetic activation of ARF6 dramatically reduced apoptosis after 48 h of vemurafenib (Fig. 4c), whereas silencing of *Arf6* significantly increased apoptosis induced by vemurafenib (Fig. S3c), consistent with a role for ARF6 in early tumor cell survival during targeted therapy. Overexpression of wildtype ARF6 also decreased vemurafenib-induced apoptosis, but to a lesser extent than ARF6^Q67L^ (Fig. 4c). Similar to ARF^Q67L^, pharmacological activation of ARF6 with QS11 almost completely abrogated vemurafenib induced apoptosis (Fig. 4d).

Combination RAF + MEK inhibition is the preferred choice of MAPKi therapy in BRAF^V600E^ melanoma patients, due to superior clinical outcomes compared to single agent RAF inhibition [47]. Thus, we interrogated ARF6 in this context. A375 melanoma cells are highly sensitive to both single-agent RAF inhibition and combination RAF + MEK inhibition in short-term cultures (Fig. S3d-e). In contrast, A2058 melanoma cells are resistant to vemurafenib (Fig. S3d), possibly due to a *MAP2K1* P124S mutation [48], but remain sensitive to the combination of dabrafenib + trametinib (Dab+Tram) (Fig. S3e). Importantly, genetic or pharmacologic activation of ARF6 reduced Dab+Tram sensitivity in these cell lines by significantly reducing apoptosis (Fig. 4e–f). These combined data suggest that the consequence of ARF6 activation upon BRAF inhibition (Fig. 4a, b and S3a) might be the emergence of resistance.

Because ARF6 activation can fortify RAF proteins (Fig. 1, S1 c, S1d, and S1i), we reasoned that ARF6 might facilitate recovery of MAPK signaling after RAF inhibition. Indeed, ARF6 activation by QS11 resulted in a markedly faster recovery of phosphorylated ERK (pERK) after vemurafenib treatment (Fig. 4g and S3f). Additional evidence that ARF6-GTP boosted MAPK recovery manifested in ERK-mediated inhibition of the apoptotic proteins BAD and BIM [5]. Unlike the control, QS11 significantly recovered ERK-mediated phosphorylation (inhibition) of BAD 24 – 48 h after vemurafenib (Fig. 4g and S3f). Furthermore, downregulation of BIM was more pronounced with QS11 (Fig. 4g and S3f). These findings demonstrate that ARF6 activation can potentiate MAPK reactivation and anti-apoptotic signaling after BRAF inhibition.

To test if ARF6-GTP promotes the emergence of DTP cells, leading to therapy resistance, we quantified colony formation during vemurafenib (Fig. 4h) or Dab+Tram treatment (Fig. 4i). Activation of ARF6 with QS11 significantly increased drug-resistant colony formation in both conditions (Fig. 4h–i and S3g-h). Hence, our overall data supports that ARF6 is activated in the early phases of adaptive resistance, acutely responding to diminished MAPK signaling, and facilitating the survival of drug-tolerant persister cells in melanoma.

### ARF6 inhibition sensitizes patient-derived, MAPK inhibitor-resistant melanoma cells

**Fig. 5:**
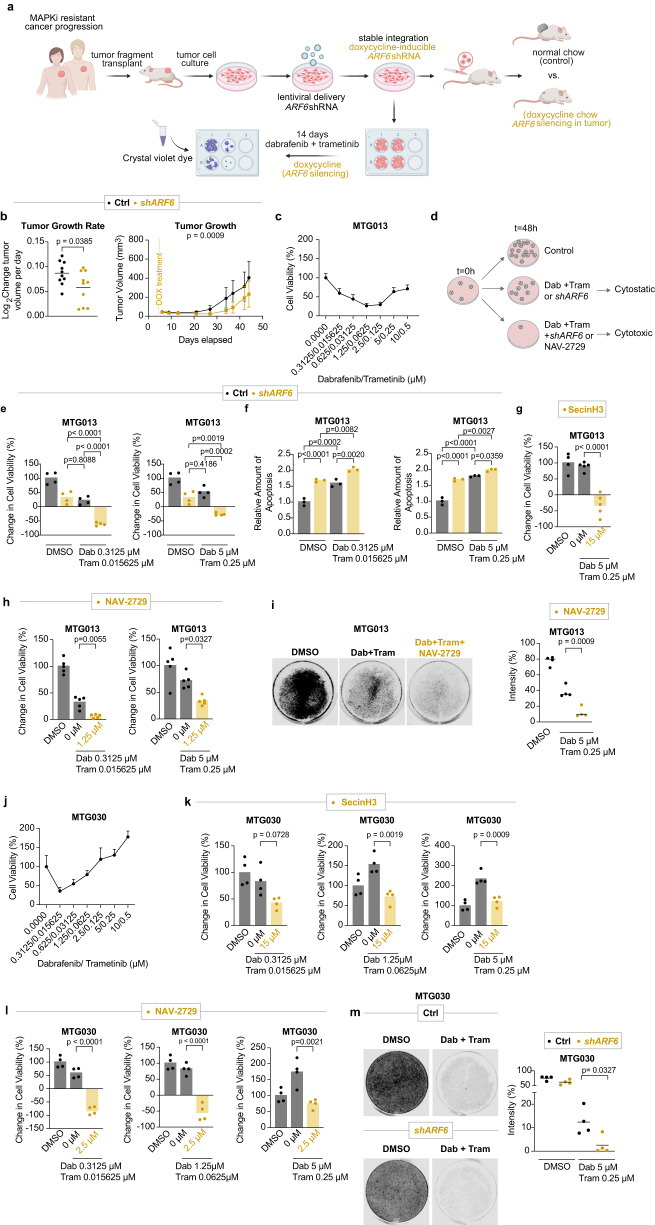
ARF6 inhibition sensitizes MAPKi-resistant cells. **a** Schematics of in vivo and in vitro experiments with patient-derived xenograft cell lines. **b** Rate of tumor growth measurements started six days after initial engraftment of MTG013 cells [stably transduced with doxycycline-induced short hairpin RNA (shRNA) for *ARF6*] in NRG mice, n = 10 controls fed regular chow, n = 10 fed doxycycline chow (*shARF6*). Tumor growth rate: two-tailed unpaired t-test with Welch’s correction. Tumor growth: Two-way ANOVA, error bars = SD. **c**, **e**, **g**, **h**, **j**, **k**, **l** Cell viability detection measured at 48 h in patient-derived cell lines (see Supplementary Table 1). **c** Dose response to Dabrafenib plus Trametinib (Dab+Tram) in MTG013, n = 5 replicates per condition, error bars = SD. **d** Schematic showing interpretation of following cell viability assays. **e**, **f** Doxycycline-induced *shARF6*. **e** n = 4 replicates per condition. **f** Apoptosis detection. n = 3 replicates per condition. **g**, **h**, **i**, **k**, **l** Pharmacologic inhibition of ARF6. **g**, **h** n = 5 replicates per condition. **i**, **m** Colony outgrowth assay in MTG013 and MTG030 for 14 days. Two-tailed unpaired t-test. **i** MTG013 treated with 5 μM Dabrafenib and 0.25 μM Trametinib ± 1.25 μM NAV-2729. n = 4 biological independent experiments. **j** Dose response of Dab+Tram in MTG030. n = 5 replicates per condition, error bars = SD. **k**, **l** n = 4 replicates per condition. **m** MTG030 treated with 5 μM Dabrafenib and 0.25 μM Trametinib, Ctrl=no doxycycline. n = 4 biological independent experiments**. e**, **f**, **g**, **h**, **k**, **l** One-way ANOVA with multiple comparisons.

Because ARF6 activation significantly reduced tumor cell death after MAPKi (Fig. 4c–f), we asked whether inhibition of ARF6 could sensitize melanoma to clinically acquired or innate MAPKi resistance. For this, we pivoted to early-passage, patient-derived xenograft (PDX) melanoma cell lines (Table S1, Fig. 5a). We recently reported that the *MET* gene is amplified in MTG013/CM013 PDX cells [49], which may explain the patient’s history of disease progression through vemurafenib treatment because HGF-MET signaling is a common mechanism of reactivation of MAPK signaling after RAFi [14]. Similar to the patient’s clinical outcome (progression through vemurafenib), MTG013 PDXs are resistant to high dose Dab+Tram [50]. We transduced these PDX cells with a doxycycline-inducible shRNA construct to conditionally knockdown *ARF6* expression after subcutaneous injection into immunodeficient NRG mice, or during in vitro colony forming assays (Fig. 5a). Doxycycline-induced knockdown of ARF6 significantly reduced tumor growth in vivo (Fig. 5b), demonstrating that ARF6 has a role in tumor progression that is independent of the ARF6-mediated adaptive immune suppression we observed in immunocompetent mice [12]. In vitro, MTG013 cells were increasingly resistant to rising concentrations of Dab+Tram (Fig. 5c), likely a result of progressive relief of an ERK negative feedback loop [5] and reactivation of MAPK signaling [51]. From these Dab+Tram dose responses, we chose a low and a high dose Dab+Tram regimen to test in combination with knockdown (Fig. 5d–f, m) or pharmacologic inhibition of ARF6 (Fig. 5g–i, k–l). Change in viability was measured over 48 h of treatment. By itself, silencing *ARF6* caused incomplete but significant loss of viability similar to Dab+Tram (Fig. 5e). Thus, inhibition of MAPK or ARF6 were equally cytostatic, but cell viability persisted above the baseline viability at time zero, indicating a low level of tumor cell survival (illustrated in Fig. 5d). Importantly, silencing of ARF6 re-sensitized MTG013 cells to Dab+Tram (Fig. 5e). Specifically, when *ARF6* knockdown was combined with Dab+Tram, there was a pronounced cytotoxic effect, where cell viability after 48 h of treatment was less than time zero, and we observed this trend with both low and high combination doses of Dab+Tram (Fig. 5e). Consistently, silencing of ARF6 increased apoptosis induced by Dab+Tram (Fig. 5f). Like genetic depletion of ARF6, prevention of ARF6 activation with the ARF6 GEF inhibitor SecinH3 [38] (Fig. 5g), or direct inhibition of ARF6 with NAV-2729 [13] (Fig. 5h), decreased viability after Dab+Tram. NAV-2729 also significantly improved sensitivity to Dab+Tram during a 14- day colony outgrowth assay (Fig. 5i). Overall, the concordance between these orthogonal methods of ARF6 inhibition demonstrates reproducible efficacy in reversing clinically acquired MAPK inhibitor resistance.

Unlike MTG013, MTG030 cells have an increased copy number of *MAP2K1* (Table S1), which encodes for the BRAF substrate and effector protein MEK1. In addition, *HRAS* is amplified. These genetic changes may explain why these PDX melanoma cells were tolerant of Dab+Tram (Fig. 5j). In fact, intermediate to high doses of Dab+Tram enhanced tumor cell viability/growth in the first 48 h of treatment (Fig. 5k, middle and right panels), and these cells appeared to be more resistant to MAPKi than MTG013 (Fig. 5c). The ARF6 GEF inhibitor, SecinH3, prevented the immediate burst in viability after Dab+Tram (Fig. 5k). Direct inhibition of ARF6 with NAV-2729 was cytotoxic when combined with low to intermediate doses of Dab+Tram (Fig. 5l, left and middle panels). Similar to SecinH3, NAV-2729 prevented the burst of enhanced viability that occurred with high dose Dab+Tram (Fig. 5l, right panel). With longer treatments (14 days), Dab+Tram reduced tumor colony formation, however, a low level of resistant tumor colonies persisted (Fig. 5m), and this was significantly diminished by knockdown of *ARF6* (Fig. 5m and S3i). Hence, these data suggest that targeting ARF6 may render melanomas with resistance mutations more vulnerable to MAPK inhibitors.

## Discussion

**Fig. 6:**
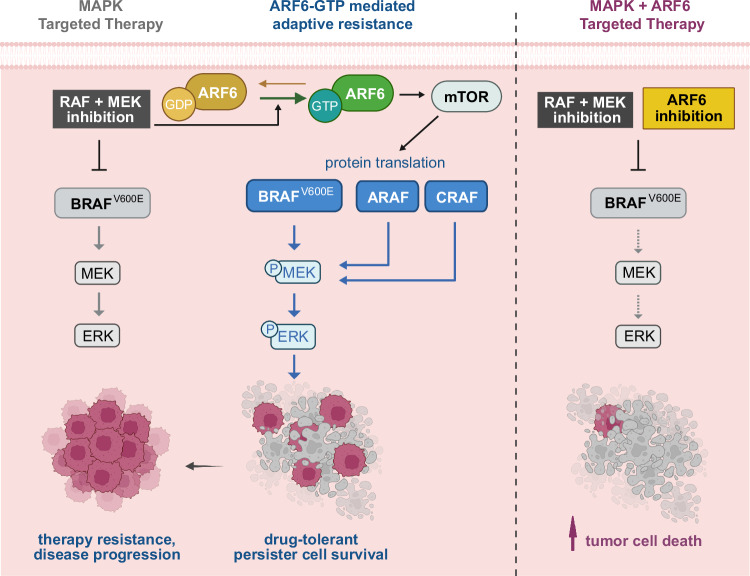
Proposed model of ARF6-dependent drug tolerant persister cell survival. Pharmacologic inhibition of BRAF^V600E^ induces ARF6 activation, triggering an adaptive stress response pathway that fortifies RAF oncoprotein synthesis, reactivation of the MAPK pathway and DTP cell survival. Combined inhibition of ARF6 and MAPK (RAF + MEK inhibition) signaling limits drug tolerance and enhances tumor cell death. Created in BioRender (2026) https://BioRender.com/6re4zlm.

We have shown that the small GTPase ARF6 helps maintain expression of BRAF^V600E^, ARAF and CRAF proteins (Figs. 1, 2 and S1c, S1d, S1g), possibly through a post-transcriptional regulatory mechanism that stimulates mTOR-dependent translation (Fig. 1j and S1g). Without ARF6-GTP, BRAF^V600E^ protein levels gradually decline in a timeline (Fig. 2c, d) that aligns with BRAF^V600E^ decay after inhibition of translation (Fig. S1e). Notably, ATP-competitive kinase inhibitors such as vemurafenib can reduce BRAF^V600E^ protein levels by preventing the HSP90 co-chaperone protein CDC37 from binding BRAF [52]. In this context, our findings suggest that cancer cells activate ARF6 in a positive feedback loop to maintain BRAF^V600E^ protein expression during kinase inhibition. Understanding how protein translation is deregulated in disease is important for the development of effective treatment approaches [53]. Messenger RNA translation through mTOR occurs in cyclical bursts in mammalian cells [54]. Thus, a dynamic cycle of activation - deactivation of ARF6 might help stimulate pulsatile surges in the synthesis of RAF kinases to maintain steady-state levels, particularly when BRAF inhibitors are present and trigger ARF6 activation. The consequence of ARF6 activation is faster recovery of MAPK signaling after RAF inhibition (Fig. 4g), enhanced survival (Fig. 4c–f) and drug-resistant growth (Fig. 4h–i). ARF6-mediated recovery of MAPK signaling may be facilitated by upregulation of ARAF and CRAF (Fig. 1c and S1g), in addition to BRAF^V600E^. While more work is needed to understand the mechanistic underpinnings and the potential extent of ARF6 regulation of protein expression, our data suggests that sustained inhibition of ARF6 can diminish BRAF^V600E^ levels and help overcome established resistance to MAPK targeted therapy (Fig. 6).

Our findings suggest that targeting ARF6 inhibits a stress-adaption pathway that gives rise to DTP cells. ARF6 mediated survival both during growth factor scarcity (Fig. 3c, d) and MAPK targeted therapy (Figs. 4 and 5). In the latter scenario, ARF6 was rapidly activated after initiation of RAFi treatment (Fig. 4a, b, and S3a) and mediated adaptive recovery of MAPK signaling (Fig. 4g). ARF6-GTP facilitated survival during the first few days of MAPKi therapy (Figs. 4c–f, 5e–h, k, l) and enabled the eventual emergence of drug-resistant growth (Figs. 4h and i, 5i, m). Overall, our data support the hypothesis that DTP cells can be drug-induced [3, 4] and provide mechanistic insights into how this phenomenon might occur in BRAF mutant cancers.

Our findings not only help explain how BRAF-mutant melanoma survives the acute phases of MAPK inhibition, they also highlight an emerging theme of pro-invasive small GTPases that link mechanisms of tumorigenesis to drug resistance. Like ARF6, the small GTPase RAC1 facilitates invasion [55], tumorigenesis [55, 56] and resistance to MAPK targeted therapy [56–58]. Recently, RAC1 was shown to be activated by MEK inhibition [55]. Interestingly, RAC1 was activated in human melanoma cells between 8 and 16 h after the initiation of treatment with trametinib. In contrast, ARF6 was activated within 1–2 h of BRAF inhibition (Fig. 4a, b). The difference in kinetics could be due to the choice of MAPKi (MEK vs. BRAF), the use of different cell lines, or possibly due to distinct upstream mechanisms that result in serial activation of these small GTPases; ARF6 followed by RAC1. Unlike ARF6, however, activation of RAC1 was not reported to signal through the MAPK pathway [56]. Like RAC1 and ARF6, RhoA also has a role in MAPKi resistance, upstream of the focal adhesion kinase (FAK)-PI3K-AKT pathway [50]. To the best of our knowledge, RAC1 and RhoA have never been shown to regulate BRAF oncoprotein expression, which may be unique to ARF6.

ARF6-dependent survival may also help explain why tumor-specific deletion of *Arf6* significantly diminished tumor development and progression in BRAF^V600E^ PTEN^WT^ melanoma models [12]. While impaired tumor formation and sluggish growth were attributable to ARF6-dependent suppression of the adaptive immune response in that model [12], our current findings suggest that ARF6 might also render tumor cells more resistant to apoptotic death incited by immune attack. More work is needed to understand ARF6-mediated tumor survival, including during immune-mediated tumor killing.

By interrogating ARF6 in vitro and in immunodeficient mice, we removed the influence of adaptive immunity and discovered an unanticipated role for ARF6 in tumor cell survival. While our findings support a mechanism whereby ARF6 activation might fortify RAF protein synthesis, translation was not directly analyzed. Other post-transcriptional or post-translational mechanisms might contribute to ARF6-mediated RAF expression. Likewise, in addition to augmenting anti-apoptotic signaling through the MAPK kinase pathway, ARF6 might stimulate other anti-apoptotic signaling pathways. For example, we have previously shown that ARF6-GTP upregulated PI3K expression and AKT-signaling in melanoma while inhibition of ARF6 reduced PI3K and AKT activation [8]. In this current study, ARF6 was critical for tumor growth acceleration caused by loss of PTEN. Together these data support that ARF6 regulates the PI3K-AKT axis and as such, it is possible that ARF6 modulates PI3K-AKT driven anti-apoptotic signaling. Lastly, because ARF6 mediates internalization [59] and recycling [60] of integrins (i.e. focal adhesion turnover), ARF6 activity might be linked to FAK-dependent resistance to MAPK targeted therapy in melanoma [50]. Independent of these possibilities, our data reveal a previously unknown vulnerability in oncogenic BRAF signaling, ARF6, which may be exploitable for addressing DTP cell survival and targeted therapy resistance.

## Methods

### Mouse husbandry, genotyping and RCAS virus delivery in vivo

Animal studies were performed in accordance with a protocol approved by the University of Utah Institutional Animal Care and Use Committee (IACUC). Generation of the *Dct::TVA*; *Braf*^*V600E*^; *Cdkn2a*^*f/f*^, *Dct::TVA; Braf*^*V600E*^; *Cdkn2a*^*f/f*^; *Arf6*^*f/f*^, and *Dct::TVA; Braf*^*V600E*^; *Cdkn2a*^*f/f*^; *Pten*^*f/f*^ murine models have been described previously [8, 12]. The flank tumor incidence, onset, growth rate and overall survival were measured and calculated as described previously [12]. Overall survival was determined at tumor size (2 cm). Mice with deep tumors (below cutaneous or subcutaneous location) were excluded from tumor onset, growth and survival analysis. Mice with tumor invasion into spine were excluded from the survival analysis due to premature termination. Investigators were blinded to *Arf6* genotype for monitoring tumor growth. Both male and female animals were used in this study and were equally distributed across experimental groups. Prior analysis confirmed that sex does not influence tumor formation, tumor size, or survival onset in our model (PMID: 39098861, PMID: 33098202).

For the PDX cell line (MTG013) model, all animal studies were approved by the University of Utah IACUC and were performed in accordance with relevant guidelines and regulations by the Huntsman Cancer Institute (HCI) Preclinical Research Resource (PRR) laboratory. 10 females and 10 males of six to eight-week-old NOD rag gamma (NGR, NOD-*Rag1*^null^ *IL2rg*^null^, NOD rag gamma, NOD-RG) mice, Jackson Laboratory stock 7799, were injected subcutaneously with 5 × 10^5^ cells in Matrigel and equally distributed across experimental groups. Mice were treated with or without Dox chow (Envigo: Global 18% Protein Rodent Diet with 625ppm doxycycline. Cat# TD.01306.) five days after injection. Mice were monitored for health weekly, and tumor size was measured twice weekly using digital calipers; the tumor volume was calculated using the following formula: (length × width^2^/2). Investigators were blinded to the chow.

### Cell lines

Authentication of all human melanoma cell lines was periodically confirmed by STR profiling in the University of Utah Genomics core facility using the Promega.

(Madison, WI) GenePrint 10 system, or by ATCC. Mycoplasma testing was performed routinely with MycoStrip® (Invivogen, Cat# rep-mysnc-50). A375, LOX-IMVI, UACC.62, were provided by Dr. M. VanBrocklin, HCI. A2058 cells were purchased from the ATCC (Cat# CRL11147D). SKMEL28 cells were provided by Dr. D. Grossman, HCI. A2058 and A375 were maintained in DMEM-high glucose (ThermoFisher Scientific, Cat# 11995073) supplemented with 10% v/v FBS (Atlas Biologicals, Cat# F-0500-DR), 1% v/v penicillin-streptomycin-glutamine (ThermoFisher Scientific, Cat# 10378016). LOX-IMVI, SKMEL2, SKMEL28, and UACC.62 cells were maintained in RPMI 1640-high glucose media (ThermoFisher Scientific, Cat# A1049101) supplemented with 10% v/v FBS, 1% v/v penicillin-streptomycin-glutamine.

Early passage, patient-derived MTG013/HCICM-013 and MTG030/HCI-CM030 melanoma cells were obtained from the HCI PRR laboratory. These primary cells were derived from tumor that was obtained from two distinct patients who provided written informed consent according to a tissue collection and usage protocols IRB 89989 and 10924, approved by the University of Utah Institutional Review Board. Access to these biospecimens is available through the HCI PRR lab. Patient-derived human melanoma cells were maintained in Mel2 media, which consists of 80% v/v MCDB 153 media (Sigma, Cat# M7403-10X1L), 20% v/v Leibovitz’s L-15 Media (ThermoFisher Scientific, Cat# 11415064), 2% v/v FBS, 1.68 mM CaCI_2_, 1x Insulin-Transferrin-Selenium-Ethanolamine (ITS-X)(Fisher Scientific, Cat# 51500056), 5 ng/mL EGF(Sigma, Cat# E-4127), 15ug/mL Bovine Pituitary Extract (ThermoFisher, Cat# 13028014), 1% v/v Penicillin-Streptomycin (ThermoFisher Scientific, Cat# 15070063).

Early passage murine tumor cell lines were derived from primary melanoma tumors induced in *Dct::TVA*; *Braf*^*V600E*^; *Cdkn2a*^*f/f*^ mice [8, 12]. Cell line 5588 = ARF6^WT^. Cell line 20000 = ARF6^NULL^. Cell line 6431 expresses ectopic ARF6^Q67L^. Cells were cultured with DMEM/ F12 HEPES (ThermoFisher Scientific, Cat # 37075) containing 10% v/v FBS, 1% v/v penicillin-streptomycin-glutamine, 1% v/v MEM Non-Essential Amino Acids Solution (ThermoFisher Scientific, Cat #11140050) under standard conditions at 37 °C in a humidified atmosphere, 5% CO_2_. DF-1 and A375-TVA cells were provided by S. Holmen (HCI). DF-1 cells were maintained in DMEM-high glucose supplemented with 10% FBS, 0.5% v/v gentamicin (ThermoFisher Scientific, Cat# 15710072), and maintained at 39 °C, with 5% CO2. A375-TVA cells were maintained in DMEM-high glucose supplemented with 10% FBS and 0.5% v/v gentamicin at 37 °C with 5% CO2 and were used to verify RCAS/Cre expression in DF-1 cells.

Human colorectal carcinoma HT-29 cells were purchased from ATCC (Cat# HTB-38) and were maintained in ATCC-formulated McCoy’s 5a Medium Modified (ATCC, Cat# 30-2007), 10%v/v FBS, 1%v/v penicillin-streptomycin-glutamine. Human glioma DBTRG-05MG cells were purchased from ATCC (Cat# CRL-2020) and were maintained in ATCC-formulated RPMI-1640 Medium (Cat# 30-2001), 10%v/v FBS, 30 mg/L L-proline (Sigma-Aldrich, Cat# 81709-10 G), 35 mg/L L-cystine (ThermoFisher Scientific, Cat# J63745.14), 3.57 g/L HEPES (ThermoFisher Scientific, Cat# 15630080), 15 mg/L hypoxanthine (Sigma-Aldrich, Cat# H9636-1G), 1 mg/L adenosine triphosphate (Sigma-Aldrich, Cat# A6419-1G), 10 mg/L adenine (Sigma-Aldrich, Cat# A2786-5G), 1 mg/L thymidine (Sigma-Aldrich, Cat# T1895-1G), and 1%v/v penicillin-streptomycin-glutamine. Cells were incubated at 37 °C in a humidified atmosphere with 5% CO_2_.

### RNA interference

Transient silencing of endogenous ARF6 was performed by sequential transfection of siRNA (*ARF6*, Qiagen Cat# 1027417; GeneGlobe SI02757286), and compared to AllStars Negative Control siRNA (Qiagen, Cat# 1027281) at a final concentration of 40 nM using Lipofectamine™ RNAiMAX transfection reagent (ThermoFisher Scientific, Cat# 13778150). Briefly, cells were seeded in a 6-well plate and first transfected with 40 nM siRNA mixed with 7.5 μL of Lipofectamine™ RNAiMAX transfection reagent. After 24 h, transfections were repeated under the same conditions. Cells were collected 24 h after the second transfection for cell viability and western blot analyses.

For conditional *ARF6* silencing with short hairpin RNA (shRNA), MTG013 and MTG030 cells were stably transduced with a replication-incompetent retrovirus (piSMART-hEF1a-GFP-shARF6, see Key Resource Table) and cultured under 1μM puromycin selection. In vitro, stably transduced cell lines were treated with 1.0 μM doxycycline.

### Western blot and ARF-GTP-pulldown

Cells were lysed using Pierce® IP Lysis buffer (ThermoFisher Scientific, Cat # 87788) with 1X Halt™ Protease and Phosphatase Inhibitor Cocktail (ThermoFisher Scientific, Cat# 78442). Protein concentrations were determined using the Pierce™ BCA Protein Assay Kit (ThermoFisher Scientific, Cat# 23227). Cell lysates were boiled with SDS sample buffer. Proteins from the cell lysates were separated by SDS polyacrylamide gel electrophoresis (SDS–PAGE) and transferred to polyvinylidene difluoride (PVDF) membranes (ThermoFisher Scientific, Cat# 88518). The PVDF membranes were blocked with TBST (10 mM Tris-HCl, 150 mM NaCl, and 0.1% v/v Tween-20) containing 5% w/v skim milk and incubated with primary antibodies. After washing in TBST, membranes were incubated with HRP-conjugated secondary antibodies and then washed with TBST before developing with Western Lightning™ Plus Chemiluminescence Reagent (PerkinElmer, Cat# NEL103001EA) or SuperSignal™ West Dura Extended Duration Substrate (ThermoFisher Scientific, Cat# 37075). Luminescent signals were detected using the Azure c300 or c600 (Azure Biosystems). Images were adjusted equally for brightness and contrast using ImageJ or Adobe Photoshop (Adobe Inc.). ImageJ (NIH, Bethesda, MD, USA) was used to quantify the intensity of bands on the blots. Relative expression was calculated by normalization to housekeeping gene used (i.e. GAPDH, tubulin) followed by the control group (e.g. DMSO, WT, etc.).

ARF-GTP pull-downs were performed using GGA3 PBD Agarose beads (Cell Biolabs, Cat# STA-419) as previously described [11]. Briefly, cells were treated with chemical compounds for the indicated time. After treatment, cells were lysed with pulldown lysis buffer (Cell Biolabs, Cat# 240102) including 1X Halt™ Protease and Phosphatase Inhibitor Cocktail. Lysates were centrifuged; supernatants were added to GGA3-conjugated beads and agitated for 1 h at 4 °C. Beads were washed in pulldown lysis buffer and prepared for western blot analysis. ARF6 and ARF1 GTP levels were interrogated with isoform-specific antibodies (see Key Resource Table).

### Cell viability assay

Cell viability was detected by CellTiter-Glo^®^ Luminescent Cell Viability Assay (Promega, Cat# G7571). Briefly, 2000 cells/well were seeded in 96 well plates overnight. The next day, cell viability was measured before treatment (0-h time point). After 48 or 72 h of treatment, media were removed and replaced with the CellTiter-Glo® Reagent. Luminescence was measured by Perkin Elmer EnVision Multi-Mode Plate Reader.

### Apoptosis assay

Apoptosis was detected by RealTime Glo™ Annexin V Apoptosis Assay (Promega; Cat# JA1000). Briefly, 10,000 cells/well were seeded in 96 well plates overnight. The next day, cells were treated with serum starvation or chemical compounds to induce apoptosis plus apoptosis detection reagent. Annexin V luminescence was measured by Perkin Elmer EnVision Multi-Model Plate Reader. Relative amount of apoptosis = mean luminescence signal intensity_EXPERIMENTAL_ t_48_ hours – t_0_ hours / mean luminescence signal intensity_DMSO_ t_48_ hours – t_0_ hours.

### Drug tolerant colony formation

Human melanoma cells A375 were seeded at 10,000 cells per well in 6 well plates and treated with vemurafenib, dabrafenib, trametinib, and/or QS11 for 30 days. For colony formation assay with early passage, patient-derived MTG013 cells were seeded at 200,000 cells per well in 6 well plates and treated with dabrafenib, trametinib, and/or NAV-2729 for 14 days. For patient-derived MTG030 cells were treated with dabrafenib, trametinib, and/or doxycycline. Drugs were refreshed every 2–3 days. After 14 or 30 days of drug treatment, cells were fixed with methanol and stained with 0.5% crystal violet stains. Plates were scanned with LICOR Odyssey^®^ DLx scanner. Colony Area or intensity was measured by ImageJ [61]. Representative images were captured with a Nikon Automated Widefield Microscope.

### Cloning, viral transduction and generation of stable cell lines

The pTRIPZ lentviral system (used for cloning pTRIPZ-ARF6^WT^-V5 and pTRIPZ-ARF6^Q67L^-V5) was gifted from Dr. Todd W. Ridky [62]. ARF6^WT^-V5 and ARF6^Q67L^-V5 were inserted into the p-TRPIZ vector using the In-Fusion Snap Assembly system (Takarabio, Cat# 638945). HEK-293T cells were co-transfected with 2nd generation lentivirus packaging vectors (5 μg pCMV-Gag/Pol, Addgene, Cat#35614; 1 μg pCMV-VSVG, Addgene, Cat# 8454) and 5 μg of expression constructs (including piSMART-hEF1a-TurboGFP-sh*ARF6*, see Key Resource Table) using Lipofectamine™ 3000 Transfection Reagent (Thermo Scientific, Cat# L3000008). Viral supernatants were harvested 48 h and 72 h post-transfection and filtered through a 0.45 μm filter. Filtered viral supernatants were applied to target cell lines together with 10 μg/ml of Polybrene (Sigma Cat# TR-1003). After infection, cells were placed in fresh media for three days before selection with 1 μM puromycin for 14 days. Doxycycline dose response treatments confirmed ectopic expression or knockdown efficiency. Stable cell lines were maintained in 1 μm puromycin.

Adenoviral ARF6^Q67L^ was created by Vector Biolabs as previously described [11]. Cells were infected with 10^7^ pfu/mL virus and incubated for 24 h prior to experimentation.

### Proteomics

Protein extraction and reverse-phase protein array of frozen mouse tumors were performed by the MD Anderson Cancer Center Functional Proteomic RPPA Core Facility.

### Quantitative reverse transcription polymerase chain reaction (qRT-PCR)

Total RNA was isolated from A375 cells after doxycycline-induced expression of ARF6^Q67L^. Cells were untreated or treated with 1 μM doxycycline for 4, 8, 24, or 48 h, then collected and stored in RNAlater (ThermoFisher Scientific, Cat# AM7024). RNA was extracted using RNeasy Plus kit (Qiagen, Cat# 74034) according to manufacturer’s instructions. Extracted RNA from each sample was converted into cDNA using SuperScript IV VILO (SSIV VILO) Master Mix (ThermoFisher Scientific, Cat# 11756050). qRT-PCR was performed in triplicate for each sample using PowerUp™ SYBR™ Green Master Mix (ThermoFisher Scientific, Cat# A25780) on the QuantStudio™ 6 Flex Real-Time PCR System (ThermoFisher Scientific) in 96-well plates. Primers used for qRT-PCR are shown in the Key Resource Table. The specificity of the amplicons was assessed by melting curve analyses. Relative mRNA expression of each gene was calculated using the number of cycles needed to reach the crossing threshold of detection (CT) and normalized to the expression of *GAPDH*.

### Immunofluorescence

Murine tumors were embedded and frozen in Tissue-Tek® O.C.T. compound. Tissues were sectioned 6–10 μm thick using a cryostat. The tissue was fixed to the slides with acetone followed by three rinses in “PBSA” (1× PBS + 0.1% sodium azide). Slides were permeabilized with 1% bovine serum albumin (BSA) + 0.1% Saponin solution, followed by blocking in PBSA + 3% v/v BSA for 60 minutes. After blocking, the slides were incubated with the primary antibody overnight at 4 °C. The next day, the slides were washed with PBSA and incubated with secondary antibody for 1 h at room temperature, then washed again with PBSA After washing the slides before counterstaining with DAPI for 30 minutes at room temperature, followed by a 5-minute wash in PBSA, and mounting in 40% w/v polyvinyl pyrrolidone + 4% v/v glycerol + 0.1% sodium azide dissolved in 1 mol/L Tris, pH 8.0. Images were collected on an Olympus Fluoview1000 scanning laser confocal microscope at 1200x magnification. Quantification of fluorescent signals on mouse tumor tissue was performed in ImageJ. Final signal intensity for BRAF, pMEK, and pERK was calculated by total green signal count divided by the number of nuclei (DAPI stained).

### Statistical analysis

Details of each statistical analysis are included in the figure legends. Statistical tests were performed using Prism software (GraphPad). Quantitative values are shown as individual sample data points or represented as the mean of at least three biologic replicates. A power analysis was performed with GPower software to estimate the number of tumor-bearing mice needed. Based on a multiple comparisons test with Bonferroni correction and a power of 0.8 and a significance level (alpha) = 0.05, we estimated we would need 25 tumor-bearing mice from each cohort to detect a 50% difference in tumor growth.

## Supplementary information

Supplemental Figure 1

Supplemental Figure 2

Supplemental Figure 3

Supplemental Table 1

Key Resource Table

## Data Availability

The data generated herein are available from the corresponding author upon request.
