# Supplementary material for: Rapid activation of ARF6 after RAF inhibition augments BRAFV600E and promotes therapy resistance
Source: Oncogene. 2026 Apr 28;45(23):2286–98. doi: 10.1038/s41388-026-03805-w (PMC13158949; doi:10.1038/s41388-026-03805-w)
Supplement: Supplementary file 1 — Supplemental Figure 1 [file 41388_2026_3805_MOESM1_ESM.pdf]

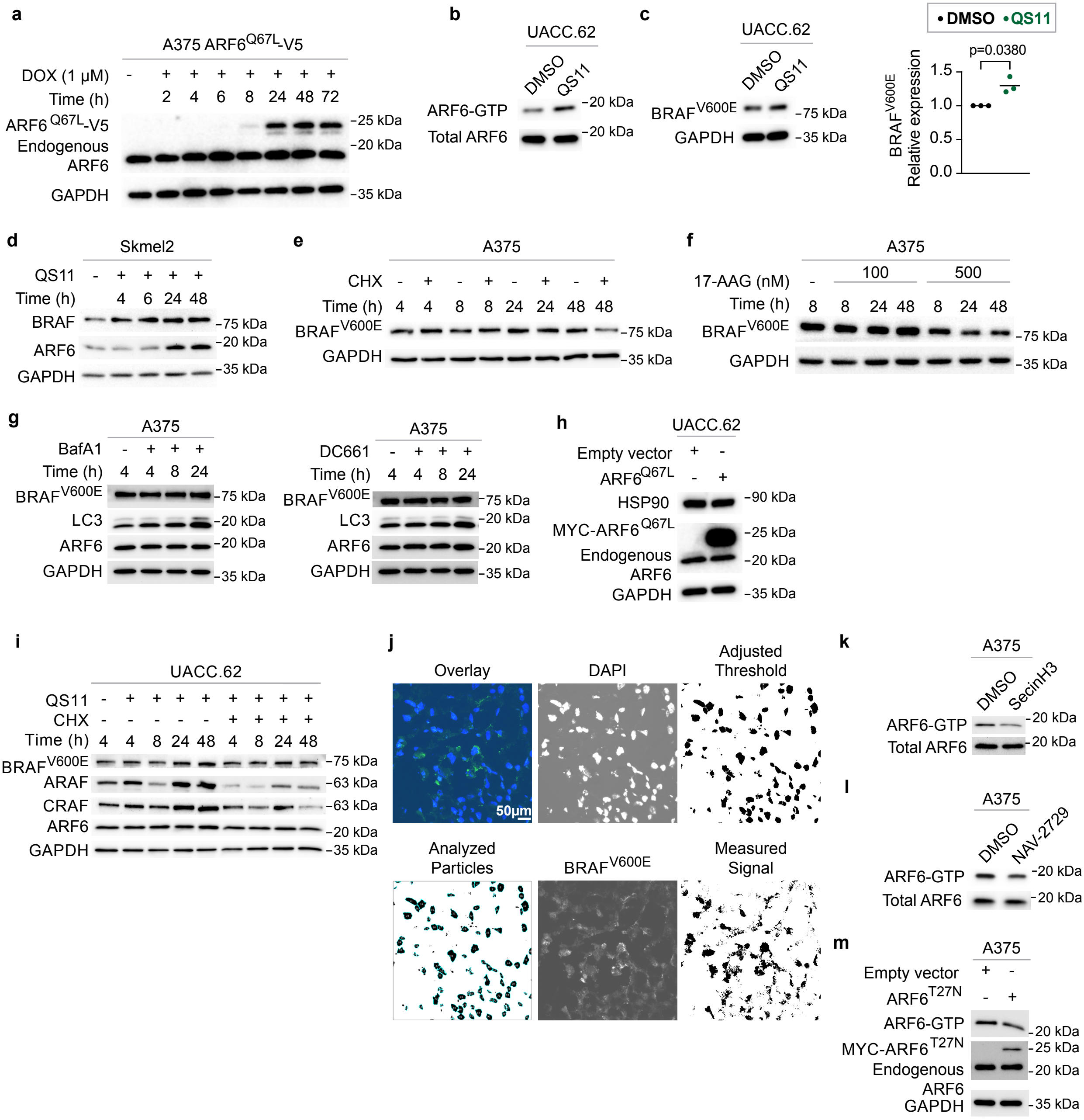

Supplementary Figure 1.

a-i, Western blot confirmation of, **a,** doxycycline (DOX)-induced ectopic ARF6 over time. **b,** QS11 (2μM)- induced increase in ARF6-GTP via ARF6-GTP pulldown following 1-hour incubation. **c,** QS11 (2μM)- induced increase in $BRAF^{V600E}$ following 24-hour incubation. P-value calculated from 3 replicates using two-tailed unpaired t-test. **d,** QS11 (4μM)- induced increase in wild type BRAF over time. **e,** Time course of $BRAF^{V600E}$ decay with cycloheximide (CHX 20μg/ml) exposure. **f,** Time course of $BRAF^{V600E}$ decay with tanespimycin (17-AAG) exposure. **g,** Stable $BRAF^{V600E}$ protein during Bafilomycin A1 (BafA1, 50nM) or DC661 (2uM) exposure. **h,** Adenoviral-delivery of ectopic, Myc-tagged $ARF6^{Q67L}$. **i,** QS11 (4μM)- induced increase in $BRAF^{V600E}$, ARAF and CRAF over time, which is diminished by CHX (20μg/ml). **j,** Representative images of ImageJ quantification steps, related to Fig. 2a. **k-m**, Inhibition of ARF6 activation, ARF6-GTP pulldowns, **k**, 3-hour incubation with SecinH3 (30μM) or DMSO. **l,** 2-hour incubation with NAV-2729 (30μM) or DMSO. **m,** adenoviral delivery of ectopic Myc-tagged $ARF6^{T27N}$. h=hours.
