# Supplementary material for: Rapid activation of ARF6 after RAF inhibition augments BRAFV600E and promotes therapy resistance
Source: Oncogene. 2026 Apr 28;45(23):2286–98. doi: 10.1038/s41388-026-03805-w (PMC13158949; doi:10.1038/s41388-026-03805-w)
Supplement: Supplementary file 2 — Supplemental Figure 2 [file 41388_2026_3805_MOESM2_ESM.pdf]

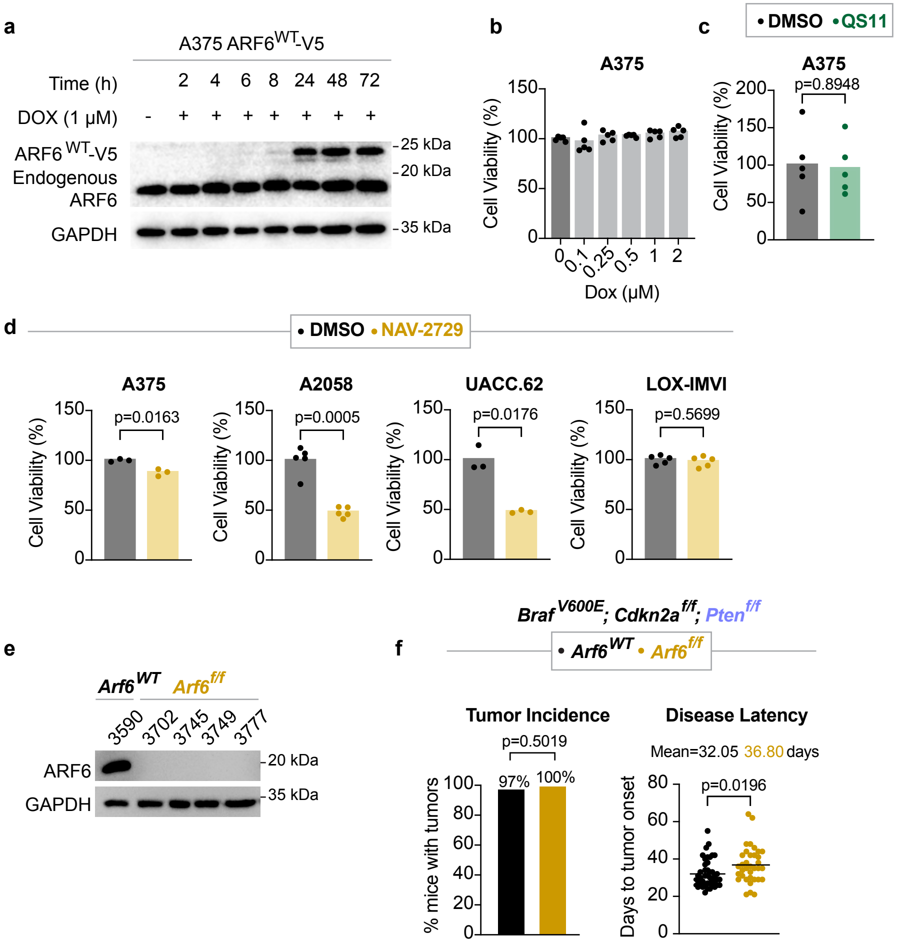

Supplementary Figure 2.

a, Western Blot confirmation of dox-induced ectopic ARF6^{WT}. **b-d,** Cell viability measured after 72 hours culture in full serum. Two-tailed unpaired t-test. **b,** Dox= doxycycline. **c,** 2μM QS11.**d,** 5μM NAV-2729.**e,** Western blot confirmation of *Arf6* deletion in tumors of *Dct::TVA; Braf*^{V600E}*; Cdkn2a*$^{f/f}$*; Pten*$^{f/f}$*Arf6*$^{f/f}$ mice. **f,** Percent of *Dct::TVA; Braf*^{V600E}*; Cdkn2a*$^{f/f}$*; Pten*$^{f/f}$mice that developed tumors within 100 days after Cre injection (tumor induction); n = 58 *Arf6* wild-type (*Arf6*^{WT}), n = 46 *Arf6* floxed (*Arf6*$^{f/f}$); two-sided Fisher's exact test. Days to initial tumor detection after Cre injection; n = 44 *Arf6*^{WT}, n = 35 *Arf6*$^{f/f}$ mice; two-tailed t-test with Welch's correction.
