# Supplementary figures and images for: Rapid activation of ARF6 after RAF inhibition augments BRAFV600E and promotes therapy resistance
Source: Oncogene. 2026 Apr 28;45(23):2286–98. doi: 10.1038/s41388-026-03805-w (PMC13158949; doi:10.1038/s41388-026-03805-w)

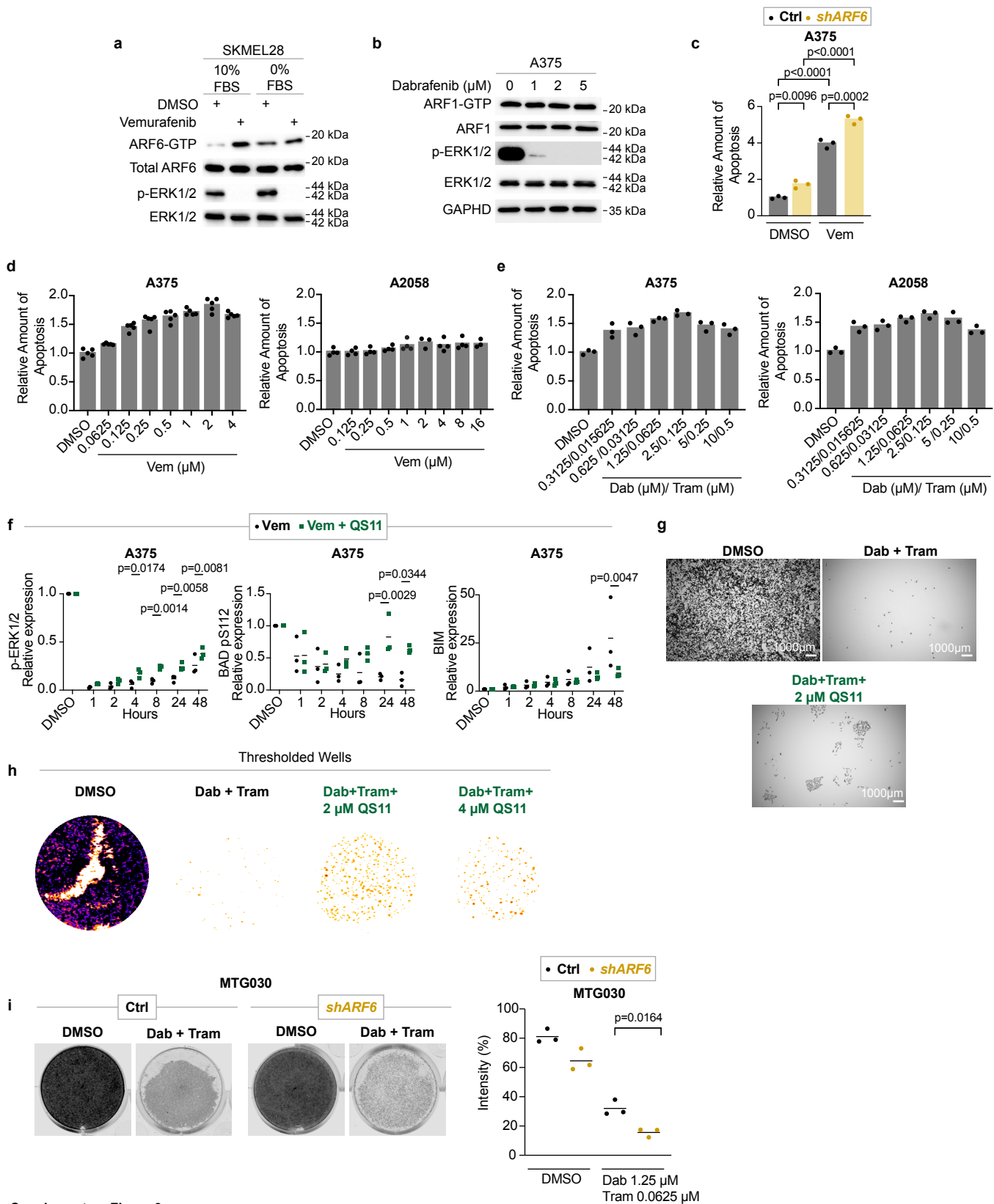

Supplement: Supplementary file 3 — Supplemental Figure 3 [file 41388_2026_3805_MOESM3_ESM.pdf]
