# Supplementary material for: Rapid activation of ARF6 after RAF inhibition augments BRAFV600E and promotes therapy resistance
Source: Oncogene. 2026 Apr 28;45(23):2286–98. doi: 10.1038/s41388-026-03805-w (PMC13158949; doi:10.1038/s41388-026-03805-w)
Supplement: Supplementary file 4 — Supplemental Table 1 [file 41388_2026_3805_MOESM4_ESM.pdf]

Supplementary Table 1

**melanoma cell line**	**tissue**	**single nucleotide variants**	**copy number variants**	**systemic therapy prior to collection**	**systemic therapy after collection**
MTG013<br>(HCI-CM013)	distant metastasis	*BRAF* c.1799T>A, p.V600E<br>*TERT* c.−146C>T (promoter)<br>*TP53* c.454C>T, p.P152S<br>*MC1R* c.478C>T, p.R160W	<u>Amplification</u><br>*MET*<br>*BRCA1,*<br>*NF1* (intragenic)<br><br><u>Deep Deletion</u><br>*PTEN*	vemurafenib	pembrolizumab
MTG030<br>(HCI-CM030)	lymph node metastasis	*BRAF* c.1799T>A, p.V600E<br>*CDKN2A* c.143C>T, p.P48L<br>*TERT* c.−124C>T (promoter)<br>*TP53* c.378C>G, p.Y126*<br>*MC1R* c.178G>T, p.V60L	<u>Amplification</u><br>*MAP2K1* (>8 copies)<br>*SPRED1* (>8 copies)<br>*HRAS*<br><br><u>Deletion</u><br>*PTEN*<br>*NF1* (intragenic),<br>*CDKN2A* (intragenic)<br>*RAF1*<br>*PBRM1*<br>*MITF*<br>*KIT*	none	pembrolizumab
