# Supplementary material for: Rapid activation of ARF6 after RAF inhibition augments BRAFV600E and promotes therapy resistance
Source: Oncogene. 2026 Apr 28;45(23):2286–98. doi: 10.1038/s41388-026-03805-w (PMC13158949; doi:10.1038/s41388-026-03805-w)
Supplement: Supplementary file 5 — Key Resource Table [file 41388_2026_3805_MOESM5_ESM.pdf]

# Key Resource Table

## Antibodies

| Target Antigen | Clone | Vendor | Catalog# | Working Dilution |
|---|---|---|---|---|
| **Antibodies (Western Blot and Immunofluorescence-Primary antibodies)** | | | | |
| **ARF6** | D12G6 | Cell Signaling Technology | 5740 | 1:1000 |
| **ARF1** | | Invitrogen | PA1-127 | 1:5000 |
| **a-tubulin** | DM1A | Cell Signaling Technology | 3873 | 1:1000 |
| **HA** | C29F4 | Cell Signaling Technology | 3724 | 1:1000 |
| **GAPDH** | D16H11 | Cell Signaling Technology | 5174 | 1:10000 |
| **ARAF** | | Cell Signaling Technology | 4432 | 1:1000 |
| **CRAF** | | Cell Signaling Technology | 9422 | 1:1000 |
| **BRAF** | EP152Y | Abcam | Ab33899 | 1:1000 |
| **BRAFV600E** | | NewEast Biosciences | 26039 | 1:1000 |
| **p-MEK1/2(S217/221)** | 41G9 | Cell Signaling Technology | 9154 | 1:1000 |
| **MEK1/2** | | Cell Signaling Technology | 9122 | 1:1000 |
| **p-ERK1/2(T202/Y204)** | D13.14.4E | Cell Signaling Technology | 4370 | 1:1000 |
| **ERK1/2** | 137F5 | Cell Signaling Technology | 4695 | 1:1000 |
| **MCL-1** | D35A5 | Cell Signaling Technology | 5453 | 1:1000 |
| **FOXO3a** | D19A7 | Cell Signaling Technology | 12829 | 1:1000 |
| **BAX** | | Cell Signaling Technology | 2772 | 1:1000 |
| **p-BAD (S112)** | | Cell Signaling Technology | 9291 | 1:1000 |
| **BAD** | | Cell Signaling Technology | 9292 | 1:1000 |
| **BIM** | C34C5 | Cell Signaling Technology | 2933 | 1:1000 |
| **β-Tubulin** | D3U1W | Cell Signaling Technology | 86298 | 1:2500 |
| **HSP90** | C45G5 | Cell Signaling Technology | 4877 | 1:4000 |
| **c-MYC** | E5Q6W | Cell Signaling Technology | 18583 | 1:700 |
| **Cyclin D1** | E3P5S | | 55506 | |
| **p-AKT(Ser473)** | D9E | Cell Signaling Technology | 4060S | 1:1000 |

| | | | | |
|---|---|---|---|---|
| **AKT** | | Cell Signaling Technology | 9272S | 1:1000 |
| **p-mTOR(Ser2448)** | | Cell Signaling Technology | 2971S | 1:1000 |
| **mTOR** | 7C10 | Cell Signaling Technology | 2983S | 1:1000 |
| **p-4EBP1(Thr37/46)** | 236B4 | Cell Signaling Technology | 2855S | 1:1000 |
| **4EBP1** | 53H11 | Cell Signaling Technology | 9644S | 1:700 |
| **LC3A/B** | D3U4C | Cell Signaling Technology | **12741** | 1:2000 |
| **V5** | D3H8Q | Cell Signaling Technology | 13202S | 1:1500 |
| **MYC** | 9B11 | Cell Signaling Technology | 2276 | 1:2000 |
| **Antibodies (Western Blot and Immunofluorescence -Secondary antibodies)** | | | | |
| **IgG** | | Jackson ImmunoResearch | 715-035-152 | 1:5000 |
| **IgG** | | Jackson ImmunoResearch | 711-035-152 | 1:5000 (1:10000 for GAPDH) |
| **Donkey anti-Rabbit IgG (H+L) Highly Cross-Adsorbed Secondary Antibody, Alexa Fluor 488** | | Invitrogen | A-21206 | 1:200 |
| **Donkey anti-Mouse IgG (H+L) Highly Cross-Adsorbed Secondary Antibody, Alexa Fluor 488** | | Invitrogen | A-21202 | 1:200 |

## Chemical Compounds

| Chemical compound | Vendor | Catalog# |
|---|---|---|
| Vemurafenib | Selleck Chemicals | 300-02 |
| Dabrafenib | MedKoo Biosciences | 205479 |
| Trametinib | MedKoo Biosciences | 201458 |
| Bafilomycin A1 | Sigma-Aldrich | SML1661 |
| QS11 | Tocris Bioscience | 3324 |
| SecinH3 | MedChem Express | HY-100559 |
| NAV-2729 | Tocris Bioscience | 5986 |
| 17-AAG | MedChem Express | HY-10211 |
| Cycloheximide | Sigma-Aldrich | C4859 |
| Torin 1 | MedChem Express | HY-13003 |
| PF-07799933 | Selleck Chemicals | E4681 |
| DMSO | ATCC | 4-X |
| Doxycycline | Sigma-Aldrich | D9891 |
| DC661 | MedChem Express | HY-111621 |

## Vectors

| Target gene | Source clone ID | Vendor | Catalog# | Gene target sequence |
| --- | --- | --- | --- | --- |
| **piSMART hEF1α/TurboGFP** | | | | |
| **ARF6** | V3IHSHEG_9625081 | Horizon Discovery | V3SH11252-229687431 | CGACCGCATCGATGAGGCT |
| | | | | |
| **Ad-CMV Null** | | | | |
| **Empty vector** | | Vector Biolabs | 1300 | |
| **Ad-CMV ARF6** | | | | |
| **Ad-ZZ2-ARF6T27N (amp)** | | Vector Biolabs | 20241217T#2 | |
| **Ad-ZZ3-ARF6Q67L (amp)** | | Vector Biolabs | 20241217T#3 | |

## Oligonucleotides

| **Oligonucleotides (qRT-PCR)** | |
| --- | --- |
| *BRAF* Forward sequence | 5’-GGCATGGATTACTTACACGCC-3’ |
| *BRAF* Reverse sequence | 5’-CTGTTCAAACTGATGGGACCC-3’ |
| *GAPDH* Forward sequence | 5’-GTCTCCTCTGACTTCAACAGCG-3’ |
| *GAPDH* Forward sequence | 5’-ACCACCCTGTTGCTGTAGCCAA-3’ |
